# A grander challenge: the case of how Makerere University College of Health Sciences (MakCHS) contributes to health outcomes in Africa

**DOI:** 10.1186/1472-698X-11-S1-S2

**Published:** 2011-03-09

**Authors:** George Pariyo, David Serwadda, Nelson K Sewankambo, Sara Groves, Robert C Bollinger, David H Peters

**Affiliations:** 1 HQ/HWA Global Health Workforce Alliance, World Health Organization, Geneva, Switzerland; 2 College of Health Sciences, Makerere University, Kampala, Uganda; 3 Johns Hopkins School of Nursing, Baltimore, Maryland 21205, USA; 4 Johns Hopkins School of Medicine, Baltimore, Maryland 21205, USA; 5 Johns Hopkins Bloomberg School of Public Health, Baltimore, Maryland 21205, USA

## Abstract

**Background:**

“Grand challenges” in global health have focused on discovery and development of technologies to save lives. The “grander challenge” involves building institutions, systems, capacity and demand to effectively deliver strategies to improve health. In 2008, Makerere University began a radical institutional change to bring together four schools under one College of Health Sciences. This paper’s objective is to demonstrate how its leadership in training, research, and services can improve health in Uganda and internationally, which lies at the core of the College’s vision.

**Methods:**

A comprehensive needs assessment involved five task forces that identified MakCHS’s contribution to the Ugandan government health priorities. Data were collected through analysis of key documents; systematic review of MakCHS publications and grants; surveys of patients, students and faculty; and key informant interviews of the College’s major stakeholders. Four pilot projects were conducted to demonstrate how the College can translate research into policy and practice, extend integrated outreach community-based education and service, and work with communities and key stakeholders to address their priority health problems.

**Results:**

MakCHS inputs to the health sector include more than 600 health professionals graduating per year through 23 degree programs, many of whom assume leadership positions. MakCHS contributions to processes include strengthened approaches to engaging communities, standardized clinical care procedures, and evidence-informed policy development. Outputs include the largest number of outpatients and inpatient admissions in Uganda. From 2005-2009, MakCHS also produced 837 peer-reviewed research publications (67% in priority areas). Outcomes include an expanded knowledge pool, and contributions to coverage of health services and healthy behaviors. Impacts include discovery and applications of global significance, such as the use of nevirapine to prevent HIV transmission in childbirth and male circumcision for HIV prevention. Pilot projects have applied innovative demand and supply incentives to create a rapid increase in safe deliveries (3-fold increase after 3 months), and increased quality and use of HIV services with positive collateral improvements on non-HIV health services at community clinics.

**Conclusion:**

MakCHS has made substantial contributions to improving health in Uganda, and shows great potential to enhance this in its new transformational role – a model for other Universities.

## Background

In 2003, the Grand Challenges in Global Health initiative was launched to promote the scientific or technological innovation that would remove critical barriers to solving important health problems in the developing world with a high likelihood of global impact and feasibility [1]. The 14 grand challenges that emerged all involve the discovery or development of technologies that would improve diagnosis, prevention, or treatment of predominantly infectious diseases prevalent in the developing world. While awaiting the promise of future technologies, others have argued that the grander challenges in global health involve overcoming a bias towards technological solutions and finding ways to ensure that delivery systems can provide technological benefits to those that need them [2,3]. Many life-saving and affordable technologies are already well known [4,5], yet many developing countries are failing to meet the Millennium Development Goals (MDGs) to improve the survival and wellbeing of children and adults in developing countries [6]. We propose that Grander Challenges in global health involve how to build the systems, institutions, innovations, and human resource capacity and demand needed to sustain the delivery of effective strategies that improve health outcomes on a large scale.

Addressing the Grander Challenges involves a greater degree of complexity than the development and dissemination of technology. The systemic factors that deliver health services or change the conditions that put people at risk of illness and premature death include ways to generate and sustain an effective health workforce, mechanisms to provide affordable, equitable, and transparent financing for health, ensuring adequate flow of information and accountability in the health sector, and finding ways for households and communities to be more empowered to make choices that affect their health. As with any complex problem, there are many entry points that can address them.

The Bill and Melinda Gates Foundation, one of the sponsors of the Grand Challenges in Global Health initiative, provided a two year learning grant to a Makerere University – Johns Hopkins University collaboration in 2009 to demonstrate how investment in a University – the Makerere University College of Health Sciences (MakCHS) – can improve health outcomes in Uganda and the East Africa region. We have formulated this opportunity as a Grander Challenge, and in this paper, report on how MakCHS is pursuing the challenge, specifically through a comprehensive needs assessment and series of strategic pilot projects.

In Uganda and other countries, it is clear that the University provides the training ground for much of the higher qualified professionals, including doctors, nurses, dentists, pharmacists, public health practitioners, and other scientists and paramedical professionals. The University is also the source of research and evaluation expertise needed to generate and assess new ideas and technologies in the health sector. The University often provides health services directly through hospitals, clinics, and public health programs. As a center of knowledge and development of the health professions, it is also a potentially important stakeholder in the policy arena in a country, influencing government and other institutions. Yet few governments and donors are willing to invest in the development of the universities in low and middle income countries, in part because they can appear to be removed from the day-to-day concerns of improving health conditions in a country, and because universities have failed to demonstrate their relevance and impact on the health sector.

The work described here reflects a new vision for the role of institutes of higher learning. The new way of doing business calls on faculty and students to systematically reassess their current roles, and how they can play critical roles in Ugandan society and internationally. The new MakCHS was started in 2008 with the intention of decentralizing authority and resources within Makerere University in order to improve efficiency and productivity, and is the first College formed at the University. The reorganization involves restructuring the existing Faculty of Medicine and Faculty of Public Health into four Schools under the direction of a College Principal, including Schools of Biomedical Sciences (e.g. anatomy, physiology, and other basic sciences), Health Sciences (e.g. nursing, dentistry, pharmacy, and other health professions), Medicine, and Public Health. The University’s vision is to be the leading institution for academic excellence and innovation in Africa. The leadership of the College of Health Sciences has used the learning grant to help forge the College as a transformational institution in Uganda and the region, influencing government, civil society and individuals, and guided by its core values of quality, professionalism, equity and social justice, relevance, accountability, and transparency. The College is motivated by its interest in improving the health of the people of Uganda and internationally through innovative and responsive teaching, learning, research and provision of services. This study puts into practice a new paradigm, involving a comprehensive needs assessment designed to align MakCHS’ educational, research and service capacity with Uganda’s national health goals and priorities, and a series of pilot projects focused on demonstrating how MakCHS can play critical roles to influence the health system in Uganda.

### The conceptual framework

**Figure 1:**
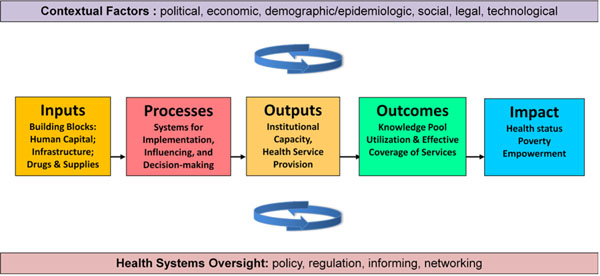
Results chain on how MakCHS contributes to better health in Uganda

In order to understand how MakCHS can improve health outcomes, we adapted a conceptual framework to model a causal pathway for how investment in the University can improve health [7]. We begin with a simplified “results chain” that is intended to show the direct effects of how MakCHS affects the inputs in the health sector and subsequently influences changes in processes, outputs, outcomes, and finally improved health status and other measures of impact in a health system (Figure 1). The inputs, or “building blocks for health systems”, involve contributions to the health workforce, physical infrastructure, drugs and supplies, and financing of health. Processes include the systems used to support health services and health research, such as methods to assure quality of health care, engage with communities, and improve management decision-making. Outputs measures refer to the delivery of health services, whether preventive, curative, or rehabilitative, and the demonstration of institutional capacity in the health sector. Outcomes refer to measures of immediate effect of the outputs in the population, such as the effective coverage of health services (e.g. vaccination coverage), and the building of the knowledge pool in health. Impact measures refer to ultimate changes in the population, such as improved health status (e.g. mortality and morbidity rates), poverty related to health, and empowerment of people to make healthy choices. A healthy population and strong health system also contribute more widely to society by raising productivity, reducing poverty, and building trust in civil institutions.

Experience has shown that simple health interventions cannot be simply replicated across countries and be expected to have the same results on improving health as they have shown in relatively controlled research environments [8]. Context and history matter greatly, as do the capabilities of individuals and organizations in a health sector, and the incentives that affect different key actors in the health sector, be they health providers, government agencies, or consumer bodies. We thus highlight the interactions between the results chain and set of contextual factors related to political, economic, epidemiologic, social, legal and technological factors, as well as the oversight or governance functions of a health system in how policy and regulation is accomplished.

## Methods

A comprehensive needs assessment was conducted by five task forces with membership from across the College to identify MakCHS’s contribution to the Ugandan government health priorities and identify strategic opportunities. The Ugandan Government’s Health Sector Strategic Plan (HSSP) is the clearest articulation of its health priorities [9,10], which defines them as the universal delivery of the Uganda National Minimum Health Care Package (UNMCP), health workforce development, and the design of evidence-informed policy and plans. HSSP disease priorities to be addressed by the UNMCP include maternal and child health, major infectious diseases (HIV/AIDS, malaria, tuberculosis), mental health, and environmental health issues.

Data were collected by the task forces through analysis of key documents; systematic review of MakCHS publications and grants; surveys of patients, students and faculty; and key informant interviews of major stakeholders of the College, with the specific methods detailed in the papers in this supplement. The specific objectives were to identify gaps and opportunities in the following areas (according to each task force):

1. **Research** to address Uganda’s current and future health sector priorities

2. **Care & service** at MakCHS, including Mulago Teaching Hospital and elsewhere in Uganda

3. **Administration & infrastructure** to facilitate effective teaching, service, and research by MakCHS

4. **Teaching & learning** at MakCHS to address Uganda’s health sector needs

5. **Stakeholders & sustainability** – MakCHS’ ability to effectively network with key stakeholders and mobilize resources to address priority health programs

Four pilot projects were also conducted to demonstrate how the College can influence the health sector to meet priority needs by:

1. How MakCHS influences policy through research (Research & Policy Pilot Project)

2. How MakCHS can work with communities to change their health systems through innovation and use of local resources (Safe Deliveries Pilot Project)

3. How to create community-University partnerships that can better serve disadvantaged populations (Kampala City Council and Gulu University Pilot Project)

4. How to provide relevant community-based education and support to health professions in under-served areas (Community Outreach Education Pilot Project)

In this paper, we look across the findings of the task forces and pilot projects, and draw upon the findings concerning the historical role played by the schools, as well as the more recent results that are emerging from the formation of the College.

## Results

### Inputs

MakCHS is Uganda’s largest health training institution and thereby a key contributor to the country’s health workforce development, producing 2,027 graduates through 23 degree programs between 2006 and 2010. The number of graduates has been increasing (692 graduates in 2010, compared to an average of 334 before that), with the largest increases due to increased enrollments in Master of Public Health and Bachelor of Environmental Health Sciences degrees. However, there are grossly insufficient numbers of students trained in order to meet HSSP priorities, with the greatest deficiencies identified in nursing. Although there may be scope for increasing the numbers of professionals trained at MakCHS, particularly if satellite campuses are developed, the task forces also recognized that there is now an important need to improve the quality of teaching at MakCHS first, before further expanding the numbers of students. For example, the task force found many positive reviews of community-based education and service (COBES), yet respondents emphasized that these programs’ administrative and educational effectiveness needs to be further improved [11]. Nonetheless, the COBES approach, which uses a problem-based learning curriculum and places students in rural settings, has already been shown to increase the willingness of graduates to work in rural areas, an important issue in Uganda and elsewhere in Africa [12]. MakCHS can also develop information communications technology and provide more technical support to other training institutions to increase the quality and number of health professionals in Uganda, both during pre-service training and for continuing education [13].

Most of the programs at MakCHS already have competency-based curricula in place that are largely relevant to goals. However, the comprehensiveness, sophistication, and mechanisms for evaluating achievement of competencies varied widely within each of the schools. Furthermore, the task force assessment revealed several important content gaps in the existing curricula, specifically, professionalism, leadership and management, communications, and ethics. Across the professions, it is recognized that graduates of MakCHS are expected to play leadership roles. For example, nearly all the District Health Officers with qualifications in public health (a requirement for the job) were educated at Makerere University. Whereas much of the training has focused on technical leadership, broader leadership and management competencies need to be emphasized in the future.

### Processes

The Needs Assessment Task Forces found many ways in which MakCHS has contributed to important processes in the health sector. College faculty have been responsible for the development of a wide variety of standard operating procedures and clinical management pathways that are used in Uganda, and in some cases, around the world. These include the clinical protocols for the prevention of mother-to-child transmission (PMTCT) of HIV [14], and hospital infection control procedures. On the other hand, the care and services task force identified that there was a lack of routine systems for improving the quality of health care at the MakCHS teaching facilities, and that patient satisfaction surveys that it conducted found high variability in patient satisfaction across hospital units.

MakCHS has also pioneered processes to empower patients and communities. The peer-to-peer approach to providing care and support for people living with HIV/AIDS was pioneered at the College, and is widely seen as improving uptake and adherence to anti-retroviral therapy across Uganda [15]. The COBES approach to community analysis and problem-solving has been found to empower communities to make better decisions about health [16]. One COBES supervisor stated that the result of the student team’s work with the community was that community members gained “the satisfaction of taking responsibility on own health”.

MakCHS faculty are increasingly involved in processes intended to inform public policies and programs. They frequently participate in government task forces, including the development of the HSSP III, proposals to the Global Fund to Fight AIDS, TB, and Malaria, and other task forces designed to develop Ministry of Health guidelines. They have also conducted timely research specifically to meet the interests of the Ministry of Health (MOH), such as the annual health sector performance assessment, analyses of the role of contracting for private, not-for-profit providers, and studies on pharmaceuticals availability. They have become involved in the design and implementation of public health programs, such as the recent rapid response to the Bududa landslide [17]. However, the collaboration between the College and the MOH is not very systematic, an issue identified by leaders in both the Ministry and the College. As one College professor opined “The MOH and MakCHS are not working hand-in-hand because of lack of institutional collaboration, as opposed to individual collaboration, and lack of planning together”. As a result of the needs assessment and strategic planning processes currently underway, plans are being put in place to more formally and systematically link the MOH and MakCHS in for planning and research.

### Outputs and outcomes

There are many health systems outputs that are directly attributable to MakCHS and its clinical and public health activities services. The Mulago Teaching Hospital is the largest provider of clinical services in the country, and by itself accounts for about 400,000 outpatient visits, 115,000 inpatient admissions, and 30,000 deliveries every year. The College’s COBES sites have involved improving health services at 43 health clinics and their surrounding villages every year, including more than 250 community assessments and 70 projects that have demonstrated improvements in community health since 2004. For example, projects have demonstrated improvements in the use of latrines, or in extending outreach primary care services as a result of COBES interventions.

MakCHS has also been influential in affecting other health institutions in the country. For example, its faculty have played pivotal roles in establishing medical schools at Gulu University and Kampala International University, and its nurses have played a leadership role in the development of the Ugandan nursing council.

**Figure 2:**
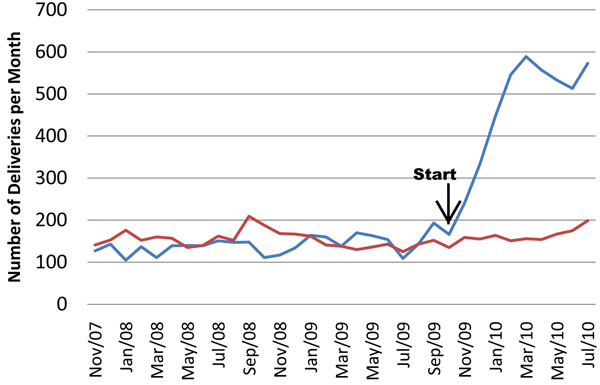
**Safe Deliveries Pilot Project: Number of institutional deliveries.** Blue line – intervention, red line – comparison. Source: [20]

The safe deliveries pilot project is another example that is showing dramatic results in health services. It is designed as a way to demonstrate how MakCHS could work differently with communities to introduce innovations that would change the way the health system works. The proportion of women having safe deliveries in Uganda has remained stubbornly low -- 41% of women nationwide had an institutional delivery in the 2006 Demographic and Health Survey [18] -- despite relatively high antenatal care coverage (94% of all women have at least one antenatal care visit). There have been many supply-side efforts to improve the training of providers and provide equipment and supplies, but a new approach was needed. Preliminary studies with community leaders, pregnant women, and health workers had indicated that poor quality of services and lack of supplies were important constraints, but also that transport and the cost of getting a delivery were prohibitive. Recognizing that motorcycle (*boda boda*) drivers were available in the community to transport women, a system was established to provide vouchers to transport pregnant women, as well as vouchers to pay health facilities additionally for each safe delivery, with the total reimbursement being around 10 USD per delivery [19]. Since the pilot project began, there has been a dramatic increase in safe deliveries in the randomly selected pilot project area, especially in contrast to the comparison area, with a 3.2 fold increase in the number of safe deliveries per month compared to the comparison area (Figure 2). There have also been marked improvements in the availability of essential supplies (e.g. gloves, cleaning solutions) and strong endorsement by community leaders and health workers. The study is ongoing, and future work will examine whether there have been changes in neonatal and maternal health.

MakCHS has also been productive in the area of health research, making important contributions to the pool of knowledge for addressing health problems in Uganda and globally. The number of peer-reviewed publications by MakCHS faculty has been growing, with over 837 publications from 2005-2009, with two-thirds of the publications lying within the priority disease conditions outlined in the current Ugandan HSSP, largely HIV/AIDS, malaria, and maternal child health [21]. However, the research task force also found that the research was almost all dependent on foreign funding, usually from the USA. Whereas the topic areas have been well aligned with Ugandan priority conditions, most of the research is clinic-based, with gaps in basic laboratory research as well as operational research to address implementation issues, and with relatively little cross-disciplinary research. Other findings of the task force showed that while 85% of faculty participate in research, only one third have been principal investigator, and only one quarter have supervised student research.

### Impact

As is the case with most health institutions and strategies, it is difficult to demonstrate a direct attribution between MakCHS and changes in morbidity and mortality at the population level. However, there are many MakCHS projects that have demonstrated improved health status and services provided that save lives at health facilities. The science that MakCHS conducts to help define a field or lay the foundation for future work is not meaningfully measured in lives saved. Past examples from MakCHS include the discovery of prostaglandins [22] and the identification of Burkitt’s lymphoma [23,24], which has been influential research that has improved the quality of life for millions of people, but whose health impact is not readily quantified. On the other hand, some research has more quantifiable health outcomes, such as the PMTCT trials pioneered at MakCHS that has led to reduced mother-to-child transmission of HIV by nearly half [14], and the male circumcision trials that reduced HIV transmission by 55% among those randomized to circumcision [25].

## Discussion

Two years is a very short time for an organization like MakCHS to learn how to define and fulfill such dramatically new vision for a college. A longer period for learning and implementing such change would likely enhance the impact of reforms, and allow for better measurement of the effects. As a result of these limitations, this study required some retrospective analysis to identify how the College has already been fulfilling some of its larger roles in society. This analysis shows that MakCHS is already playing a pivotal role in contributing to health systems, through each step of the results chain to better health. It is clear that MakCHS’s teaching, research, and service provision functions align well with the Ugandan government’s disease priorities, as MakCHS has been clearly prioritizing major infectious diseases and maternal and child health. However, it is also clear that mental and environmental health issues are less well addressed.

The task forces also identified a large range of deficiencies that would need to be addressed for MakCHS to better play an even more effective role in the health sector, including more stable sources of funding to develop critical systems for grants management, infrastructure development, and to ensure that faculty and student incentives are aligned with the mission of the College. There are currently few opportunities for institutions like the MakCHS to obtain additional core funding from domestic governments, and foreign sources of funding are almost exclusively tied to specific projects, and do little to cover the core costs of an institution. However, core funding to build institutional capacity is exactly what is needed by institutions like MakCHS.

The way in which this study was conducted demonstrates a new way for the College to do its business. Each of the task forces is an inter-disciplinary team, comprised of members of each of the four Schools. The work was done in a systematic way, and designed to show accountable results to multiple stakeholders, and relevance to the health system in Uganda and the region. In the future, further multi-disciplinary research and educational approaches will be pursued. For example, the new masters degree in nursing will involve partnership with the School of Medicine for training and clinical practice. Similarly, the departments of nursing and pharmacy will jointly conduct community outreach in screening for hypertension. A new Medical Education Partnership Initiative grant will involve all the Schools at MakCHS to develop multi-disciplinary community-based platforms for education, service, and research across Uganda, in collaboration with other medical schools and other key stakeholders in Uganda.

## Conclusions

The College has a strong and continuing history of making substantial contributions to improving health in Uganda. It also shows great potential to enhance this by taking on its new transformational role, which can be a model for other Universities. As the MakCHS now develops its strategic plan to outline its long term future, it is clear that it will have to face many uncertainties. Nonetheless, the work reported here has already helped to define a future direction that will focus on improving quality in teaching and learning, research, and services. The process has already enabled the College to work more closely with the MOH, and to revise curricula and the way health services are provided. It has also helped MakCHS put together a coherent plan for support of the US National Institutes of Health (NIH) Medical Education Partnership Initiative. Other specific plans will be devised to overcome weaknesses in management systems and infrastructure, but also to engage faculty and students with key stakeholders in Uganda, beginning with MOH and civil society, to promote innovation and evaluation. With additional support to carry out its vision as a transformational institution, MakCHS can become an even stronger model for taking on the grander challenge of building the systems to accelerate and sustain better health in Uganda and elsewhere.

## Competing interests

The authors declare that they have no competing interests.

## List of abbreviations used

MakCHS: Makerere University College of Health Sciences; MDGs: Millennium Development Goals; HSSP: Health Sector Support Plan (Uganda); UNMCP: Uganda National Minimum Health Care Package; COBES: Community Based Education and Service; PMTCT: Prevention of Mother-to-Child Transmission; MOH: Ministry of Health; NIH: National Institutes of Health (United States).

## Authors' contributions

DHP, DS, RCB, and NKS participated in the design of the study, while DHP and GP developed the conceptual framework. DHP, DS, GP, NS, SG and RCB participated in the analysis, writing, and review of the manuscript. All authors read and approved the final manuscript.
